# Interface‐Mediated Twinning‐Induced Plasticity in a Fine Hexagonal Microstructure Generated by Additive Manufacturing

**DOI:** 10.1002/adma.202105096

**Published:** 2021-10-19

**Authors:** Pere Barriobero‐Vila, Juan Manuel Vallejos, Joachim Gussone, Jan Haubrich, Klemens Kelm, Andreas Stark, Norbert Schell, Guillermo Requena

**Affiliations:** ^1^ Institute of Materials Research German Aerospace Center (DLR) Linder Höhe 51147 Cologne Germany; ^2^ Rosario Physics Institute National University of Rosario CONICET‐UNR Bv. 27 de febrero 210 bis Rosario Santa Fe 2000 Argentina; ^3^ Helmholtz‐Zentrum Hereon Max‐Planck‐Straße 1 21502 Geesthacht Germany; ^4^ Metallic Structures and Materials Systems for Aerospace Engineering RWTH Aachen University 52062 Aachen Germany

**Keywords:** deformation twinning, hexagonal close‐packed alloys, in situ high‐energy synchrotron X‐ray diffraction, metal 3D printing, structural properties

## Abstract

The grain size is a determinant microstructural feature to enable the activation of deformation twinning in hexagonal close‐packed (hcp) metals. Although deformation twinning is one of the most effective mechanisms for improving the strength–ductility trade‐off of structural alloys, its activation is reduced with decreasing grain size. This work reports the discovery of the activation of deformation twinning in a fine‐grained hcp microstructure by introducing ductile body‐centered cubic (bcc) nano‐layer interfaces. The fast solidification and cooling conditions of laser‐based additive manufacturing are exploited to obtain a fine microstructure that, coupled with an intensified intrinsic heat treatment, permits to generate the bcc nano‐layers. In situ high‐energy synchrotron X‐ray diffraction allows tracking the activation and evolution of mechanical twinning in real‐time. The findings obtained show the potential of ductile nano‐layering for the novel design of hcp damage tolerant materials with improved life spans.

## Graphical abstract

Deformation twinning is one of the most effective mechanisms for improving the strength–ductility trade‐off of structural alloys. However, its activation in hexagonal‐close‐packed (hcp) metals is reduced with decreasing grain size. This work reports the discovery of the activation of deformation twinning in a fine‐grained hcp microstructure obtained by laser‐based metal additive manufacturing.

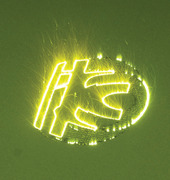

## 1 Introduction

The plasticity of metals relies on the activation of microscopic deformation mechanisms such as dislocation slip or mechanical twinning.^[^ ^1^ ^]^ Hexagonal close‐packed (hcp) metals like Ti, Zr, and Mg, are used in high‐value engineering applications including the aerospace, nuclear, automotive, chemical engineering, and bioengineering sectors.^[^ ^2^ ^]^ Ti‐alloys find a widespread use in aerospace structural components such as fan blades of jet engines or landing gears. They exhibit superior specific strength (yield strength/density) than other structural alloys within a broad range of service temperatures, and present excellent corrosion resistance up to ≈500 °C.^[^ ^3^ ^]^

Mechanical twinning plays a very relevant role during the plastic deformation of hcp metals owing to the insufficient number of independent slip systems that are usually active in hcp lattices. In these materials, <a> slip does not provide the necessary five degrees of freedom required for an arbitrary shape change, and only in particular cases, deformation may be accommodated via <c+a> slip.^[^ ^2^, ^4^ ^]^ This anisotropy of the hcp lattice usually leads to difficult processing routes and a cautious use for high‐performance structural applications.^[^ ^2^ ^]^ In addition to slip, the activation of mechanical twinning has been exploited in Ti, Mg, and Zr alloys for accommodating plastic deformation.^[^ ^5^, ^6^, ^7^ ^]^

Twinning‐induced plasticity (TWIP) is one of the most effective mechanisms for improving the strength‐ductility trade‐off. The design of metals that present a simultaneous increase in these properties has been an essential goal of metallurgical research.^[^ ^4^, ^8^ ^]^ During TWIP, the formation of twin interfaces creates a barrier for the propagation of dislocations, leading to a Hall–Petch‐type grain size effect, and a consequent strain hardening. The presence of twins reduces the mean free path of dislocations that result in an effective barrier to dislocation motion.^[^ ^9^ ^]^

The grain size is a determinant microstructural feature enabling the activation of deformation twinning. Most of the investigations on deformed hcp metals as well as polycrystalline metals with other crystal lattices, reported that the propensity of deformation twinning is reduced as the grain size decreases, when considering analogous crystal orientations. This effect has been successfully used to suppress or lower the formation of twinning via grain refinement.^[^ ^5^, ^10^, ^11^ ^]^

Plentiful studies on hcp alloys showed that mechanical twinning is promoted within coarse grains (>1 µm) during deformation.^[^ ^5^, ^6^, ^7^, ^9^, ^10^, ^11^, ^12^, ^13^ ^]^ However, its propagation and exploitation in fine‐grain microstructures received less attention owing to the much more difficult activation of this deformation mechanism.^[^ ^14^ ^]^ Also, it has been proved that twinning is suppressed in TWIP steels and Cu alloys with a reduction in grain size.^[^ ^15^ ^]^

In addition to the grain size, the twinning behavior of hcp metals depends on the c/a ratio and compositional factors associated with particular stacking fault energies. Also, the occurrence of deformation twinning depends on deformation conditions, such as strain, strain rate, temperature, as well as microstructure features such as the preferential crystallographic texture and the grain size.^[^ ^2^, ^10^, ^11^, ^12^ ^]^

In this work, we report the discovery of the activation of deformation twinning in the fine‐grain microstructure of a Ti‐6Al‐4 V alloy by taking advantage of grain interfaces and crystal orientation. Laser‐based additive manufacturing (LAM) is used to create a fine microstructure of hcp grains decorated by nanometric and ductile body‐centered cubic (bcc) interfaces. Compared to traditional manufacturing, the rapid solidification conditions and fast cooling of LAM allow for the creation of microstructures with small grain size.^[^ ^16^ ^]^ The results obtained show that twins can propagate during deformation through sub‐micron α grains. This is facilitated by the introduction of ductile β nanolayers.

Since the critical resolved shear stress (CRSS) for <c+a> slip is comparable to that for twinning in hcp metals including Ti and Zr,^[^ ^2^ ^]^ both mechanisms are usually simultaneously active. This complicates the interpretation of deformation data and makes the determination of the twinning kinetics a difficult task. Also, the activation of mechanical twinning is hard to capture experimentally owing to the fast processes involved and the complex interplay of very fine microstructural features. Consequently, most of the research has focused on characterizing these processes by transmission electron microscopy (TEM) of post‐mortem samples.^[^ ^2^, ^4^ ^]^ Nowadays, time‐resolved techniques available at synchrotron radiation sources provide unique insights into the deformation kinetics necessary to understand the competition between slip and twinning. Compared to post‐mortem approaches, these methodologies provide real‐time visualization of the extent of these mechanisms avoiding biased interpretations.

In this work, in situ high energy synchrotron X‐ray diffraction (HEXRD) is employed to determine the activation sequence of bulk deformation mechanisms. This technique allows for a continuous tracking of the deformation kinetics. Although twinning‐induced plasticity is an unusual deformation mechanism for the studied Ti–6Al–4V alloy,^[^ ^17^ ^]^ in situ HEXRD provides a univocal evidence of the contribution of mechanical twinning to deformation. Consequently, a high strength‐ductility trade‐off is obtained compared to the state‐of‐the‐art materials.

These findings show the potential of ductile interface nano‐layering for promoting mechanical twinning in small grain microstructures and open up new TWIP‐based alloy‐design strategies for damage tolerant materials.

## 2 Results

The insufficient structural performance of as‐built LAM Ti‐alloys is well known to occur in the popular Ti–6Al–4V alloy,^[^ ^18^ ^]^ which accounts for more than 50% of the titanium market and leads —by far— the additive manufacturing of Ti alloys.^[^ ^19^ ^]^ On the one hand, martensitic microstructures with reduced ductility (<14%) are usually obtained by the LAM technique laser powder bed fusion (LPBF) owing to the fast cooling rates achieved (>10^3^ °C s^−1^). Compared to stable α−β structures, the lower ductility and fracture toughness exhibited by martensitic α′ microstructures are mainly a consequence of high density of defects present in the α′ phase (e.g., dislocations) and the lack of the softer bcc β (α and α′ have a hcp lattice).^[^ ^20^, ^21^ ^]^ This differs from the brittle martensite in steels consisting of a distorted bcc tetragonal lattice with interstitial C atoms.^[^ ^22^ ^]^

**Figure 1:**
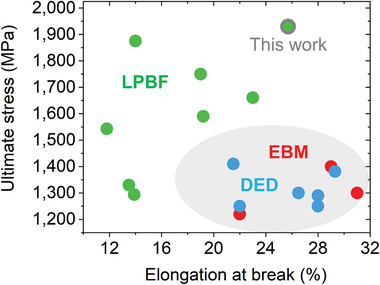
Increase of ductility at expenses of strength for laser‐based additive manufacturing (LAM) Ti–6Al–4V. This work investigates a LAM condition outperforming this property trade‐off due to a dominance of mechanical twinning over slip during deformation. Values of ultimate strength and deformation at break obtained during uniaxial compression of Ti–6Al–4V produced by the following laser‐based additive manufacturing methodologies: laser powder bed fusion (LPBF), direct energy deposition (DED), and electron beam melting (EBM)^[^ ^23^, ^24^, ^25^, ^26^, ^27^, ^28^, ^29^, ^30^, ^31^, ^32^, ^33^, ^34^, ^35^, ^36^, ^37^ ^]^ –shown in green, blue and red, respectively. The condition studied in this work is surrounded by a bold line.

On the other hand, most of the strategies to improve the ductility of LPBF Ti–6Al–4V have been focused on inducing the decomposition of the α′ martensite into lamellar α+β microstructures. Such decomposition is pronounced during LAM manufacturing by direct energy deposition (DED) and electron beam melting (EBM) owing to the lower cooling rates involved and longer exposure times at high temperature.^[^ ^18^ ^]^ This leads to higher fractions of the bcc β phase which are more ductile than the hcp α phase, as well as to coarser microstructures compared to those obtained by LPBF. However, this approach alone leads to an increase of ductility at expenses of a considerable reduction of strength (see **Figure** **1**). It is important noting that when comparing, for example, the EBM process to vacuum annealing treatments, the fraction of β may not present remarkable differences.

Figure 1 shows a literature review of the ultimate stress and deformation at break obtained during uniaxial compression of Ti–6Al–4V produced by the LAM methodologies LPBF, DED, and EBM.^[^ ^23^, ^24^, ^25^, ^26^, ^27^, ^28^, ^29^, ^30^, ^31^, ^32^, ^33^, ^34^, ^35^, ^36^, ^37^ ^]^ The values provided result from compression testing and a tension‐compression asymmetry should be expected. Though this data allows comparing the strength–ductility trade‐off between conditions, it is important to remark that industrial applications of Ti–6Al–4V (e.g., aerospace) require considering further performance relevant under service conditions including high and low cycle fatigue properties. As pointed out above, EBM and DED usually provide more ductile as‐built conditions at the expense of strength while an inverse trend is observed for LPBF conditions. As shown in Figure 1, the case analyzed in this work obtained by LPBF provides a superior trade‐off owing to the activation of TWIP. This effect, discussed in the next lines, leads to an ultimate stress of 1930 MPa and an elongation at break of 26%. The obtained stress–strain curve is provided in Figure S1, Supporting Information. This figure shows that the evolution of the strain hardening rate is characterized by the periods of decrease, stabilization, and second decrease during strain <0.05, 0.05−0.22, and >0.22, respectively. The strain hardening rate presents a steady plateau in the intermediate stage of deformation (strain = 0.05–0.18) which corresponds to a type of strain hardening rate curve reported for TWIP Ti‐alloys.^[^ ^38^, ^39^ ^]^ Three regimes of strain hardening also take place in commercially pure titanium during deformation twinning.^[^ ^40^ ^]^

**Figure 2:**
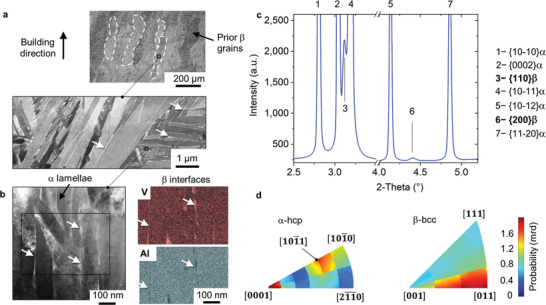
Laser powder bed fusion (LPBF) allows for the creation of microstructures with fine α grains and β‐nano‐layers at the interfaces. Alloy before deformation: a,b) scanning electron microscopy (SEM‐BSE) images and transmission electron microscopy (bright field and energy‐dispersive X‐ray spectroscopy, EDX, of the selected inset). c) diffraction pattern of the bulk material (gauge volume 1 × 1 × 3 mm^3^) and d) inverse pole figures obtained for the compression direction using high energy synchrotron X‐ray diffraction (HEXRD).

**Figure** **2**a shows the microstructure of the studied as‐built LPBF Ti–6Al–4V alloy before deformation. It consists of columnar prior‐β grains oriented along the LPBF building direction whose boundaries are highlighted by dashed lines. Within these prior‐β grains, the microstructure consists of lamellar α + β arrangements formed by packets of fine hcp α lamellae of a mean thickness ≈100–500 nm (Figure 2a,b). As pointed out by the arrows in Figure 2b, they are separated by thin continuous β layers of <50 nm in thickness that are rich in V and poor in Al. The microstructure obtained is a consequence of partial decomposition of α′ into α + β, correlating well with previous investigations reporting this effect using high energy densities during LPBF.^[^ ^41^ ^]^ Thus, the hcp grains of the microstructure can include α′ and α phases. For simplicity, they will be referred onwards as α.

The diffraction pattern in Figure 2c, obtained by HEXRD, shows mainly peaks of hcp α. In addition, reflections corresponding to bcc β can be observed. The formation of this phase is a consequence of the intensified intrinsic heat treatment (IHT) driven by diffusive martensite decomposition.^[^ ^42^ ^]^ The LPBF scanning strategy used combines porosity‐optimized processing with a very tight hatch distance promoting the IHT with long exposure periods of the alloy to high temperatures. Fine β layers form as a consequence of IHT at temperatures at which diffusion is active below the martensite start temperature (*M*_s_) of ≈800 °C for Ti–6Al–4V. Considering that at T < *M*_s_ formation of martensite can be suppressed upon fast cooling, successive precipitation of β takes place instead, owing to the decreasing influence of the effective IHT temperature during further manufacturing. During IHT in the α + β field, element partitioning results in the diffusion of V to β, and Al to α.^[^ ^42^, ^43^ ^]^

The phase fraction of β obtained by quantitative Rietveld analysis is 6.7 ± 2 vol%. A composition range of 10−20 at% V is expected within β according to our previous investigations using atom probe tomography.^[^ ^43^ ^]^

The inverse pole figures in Figure 2d from the bulk material (gauge volume 1 × 1 × 3.5 mm 3) show the initial texture prior deformation for the compression direction. α presents a preferential orientation of the [0001] *c*‐axis parallel to the compression direction (C). A less pronounced component for the direction in refs. [10, 11] can be observed. On the other hand, the observed texture of [011]β parallel to C denotes that this phase presents a Burgers orientation relationship (OR) (0001)α || (011)β with the preferential (0001)α orientation. In addition, the pole figures for {0002}α and {110}β are presented for this condition in Figure S2, Supporting Information.

**Figure 3:**
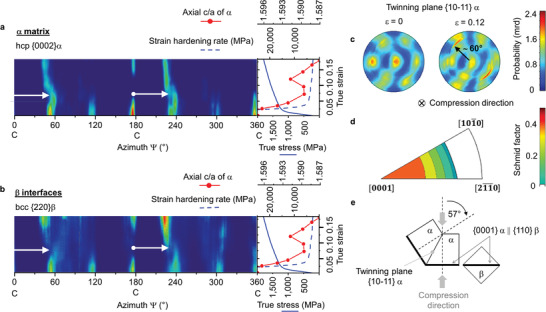
Transmission of twinning through the β interfaces. Time‐resolved tracking of deformation kinetics using in situ high energy synchrotron X‐ray diffraction (HEXRD). Color‐coded 2D plots corresponding to the evolution of a) {0002}α and b) {220}β for the azimuthal range 0–360° during uniaxial compression of the Ti–6Al–4V alloy fabricated by LPBF. The corresponding curves for the evolution of true stress–strain, strain hardening rate, and c/a ratio in the axial (or deformation) direction of the α hcp lattice are shown on the right side of the 2D plots. c) Pole figures of {10‐11}α obtained from the bulk material by in situ HEXRD (gauge volume 1 × 1 × 3.5 mm^3^) for the initial condition (ε = 0) and during uniaxial compression up to ε = 0.12. d) Schmid factors for the {10–11} <10–1–2> compression twinning system. e) Schematic diagram representing the reorientation of the hcp lattice associated with compression twinning.^[^ ^2^ ^]^

**Figure** **3**a shows the evolution of the basal {0002}α reflections as a function of strain obtained by in situ HEXRD during uniaxial compression. As shown in Figure 2d, this reflection is representative of the sample's main texture component. The compression direction, C, indicated in the horizonal azimuth axis, is perpendicular to the sample's LPBF building direction. The stress–strain curve is shown on the right side of the 2D color‐coded plots. Initially, the maximum intensities are separated by ≈60° along the azimuth axis. This reflects the texture associated with the hcp variants obtained after LPBF. During deformation up to ε ≈ 0.05, {0002}α reflections located in the compression direction (indicated by “C” for the azimuth angles ≈0/360° and ≈180°) gradually vanish. Simultaneously, new reflections become visible at ≈55° and ≈235°. The arrows in Figure 3a indicate an apparent correlation between the vanishing and new reflections as a sudden shift of ≈50–60° of the hcp basal planes {0002} from the compression direction. These sudden shifts are related to rapid rotations of a significant fraction of the crystals due to compression twinning {10–11} <10–1–2> (rotation angle = 57°). In addition, the diagram at the right of Figure 3a shows that mechanical twinning is associated with an increase of the c/a ratio from 1.589 up to 1.591 between ε ≈ 0.05–0.12 indicating a relaxation of the hcp lattice in the compression direction.

The activation of the twinning system {10–11} <10–1–2> correlates well with the pole figures of the twinning plane {10–11}α shown in Figure 3c. During compression up to ε = 0.12, Figure 3c shows new poles forming at ≈60° with respect to the compression direction. This figure reflects the stereographic projection of the normal to the twinning plane. These poles form as a consequence of new grains forming according to the twinning orientation. Such reorientation of the hcp crystal structure during twinning is represented schematically in Figure 3e. The directional distribution of the poles shown in Figure 3c is probably a consequence of twinning by crystals that in the initial condition present slightly different misorientations around the *c*‐axis.

The Schmid factors calculated for the {101–1} <10–1–2> compression twinning system (Figure 3d) show that in the studied as‐built LPBF alloy, the basal orientations are the most prone to activate this mechanism when oriented perpendicularly to the compression direction. Figure 3b shows the evolution of {220}β reflections. According to the Burgers orientation relationship, this plane is parallel to the one shown in Figure 3a. A remarkable finding is that the bcc β interfaces undergo a rotation analogous to that of α during twinning. This effect points to a twinning transfer across α grains mediated by the β interfaces.

**Figure 4:**
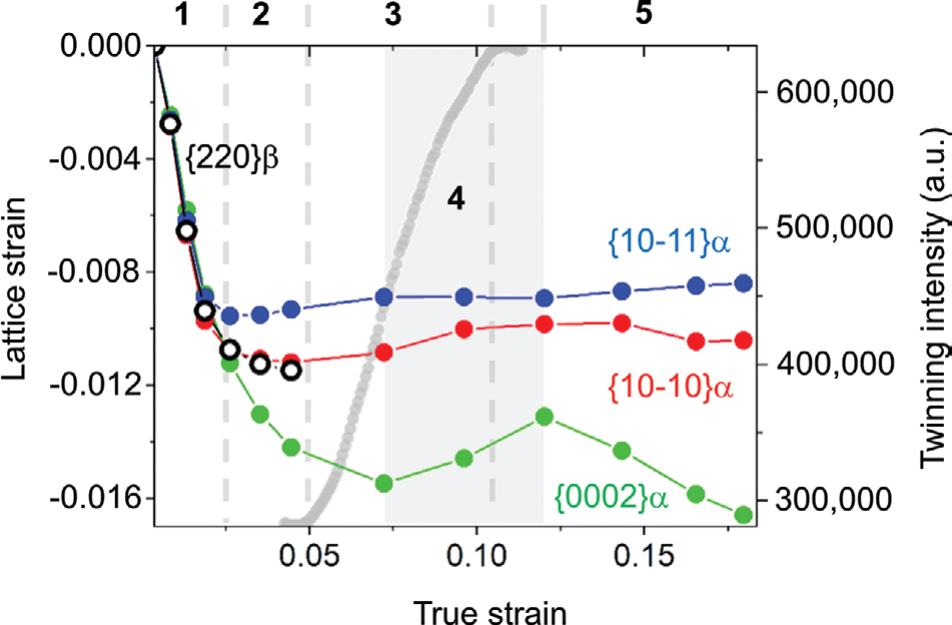
Deformation stages: 1) elastic regime, 2) slip activation, 3) twinning formation, 4) relaxation of the matrix (deformation dominated by twinning), 5) deformation dominated by slip. Evolution of the twinning intensity (grey curve) and lattice strains obtained for the hcp {0002}α, {10‐10}α, and {10‐11}α planes, as well as for the bcc {220}β plane in the compression direction during deformation. The error bars are comprised within the symbols.

**Figure** **4** shows the evolution of the axial lattice strains for the basal {0002}α, the pyramidal {10‐11}α, and prismatic {10–10}α planes in the compression direction. In addition, the evolution of the twinning intensity obtained from the intensity timelines presented in Figure 3a is plotted as a function of strain. The following deformation stages are identified (Figure 4):

1) Elastic regime (ε ≈ 0–0.025): it comprises the deformation period up to yield point (see diagram on the right side of Figure 2a,b) characterized by a steep decrease of all lattice strains.
2) Slip activation (ε ≈ 0.025–0.05): the lattice strains {10–11}α, {10–10}α, and {220}β remain nearly constant. This yield indicates the onset of plastic deformation for these orientations. {220}β reflections are not shown at higher strain because a severe decrease of intensity in the compression direction hinders its analysis.
3) Twinning formation (ε ≈ 0.05–0.11): the intensity of additional reflections associated with twinning increases. These are indicated by arrows in Figure 3a. The decrease in the change of rate for {0002}α in stages 2 and 3 is a consequence of the activation of slip for this orientation (main texture component).
4) Relaxation of the α matrix (ε ≈ 0.075–0.125): the lattice strain {0002}α undergoes a significant increase from −0.0154 to −0.0131 due to the relaxation of the matrix as new twins are formed. This reflects that the stress along the loading axis decreases during compression in a major fraction of the grains with the c‐axis oriented in the compression direction. The activation of a twin system is expected to induce stress relaxation in the parent grain. A stress relaxation inside grains undergoing twinning was reported for {0002}α orientations during compression of hcp Mg.^[^ ^44^ ^]^ Additionally, the lattice strains of {10–10}α and {10–11}α present a slight increase. These features point to a deformation dominated by twinning in this stage although slip is also active since stage 2.
5) Deformation dominated by slip (ε > 0.125): the lattice strain for the main texture component {0002}α undergoes a second decrease after the formation of twinning and relaxation of the parent matrix, while {10–10}α and {10–11}α remain constant.

## 3 Discussion

**Figure 5:**
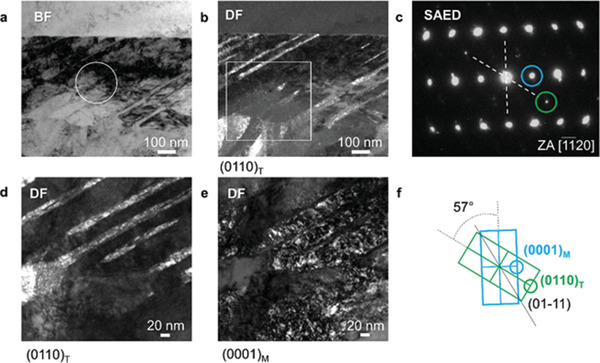
Nano‐twins within the fine‐grain hcp microstructure after deformation. Transmission electron microscopy (TEM) of the LPBF Ti–6Al–4V alloy after uniaxial compression at room temperature. a) bright field (BF) image of α lamellae and the corresponding b) dark field (DF) image showing the presence of nano‐sized twins in their interior. c) Selected area electron diffraction (SAED) pattern of the [–1–120]α zone axis (ZA) taken from the encircled region in (a). d) Higher magnification DF image of the framed region in (b) and e) the corresponding image from the matrix using the reflections encircled in green and blue in (c), respectively. f) Schematic diagram of the diffraction pattern in (c). The reflections of the matrix and the twin are indicated as M and T, respectively.

The activation of twinning during deformation is also shown by microstructural analysis using transmission electron microscopy (TEM) in **Figure** **5**. Here, the dark field (DF) images of Figure 5b,d show the presence of nanotwins through hcp α grains after deformation. Both HEXRD and TEM point to the occurrence of {10–11} <10–1–2> compression twinning associated with a hcp lattice rotation of ≈57° (see Figures 3a and 5f). This represents the system with the lowest twinning shear for hcp titanium.^[^ ^2^, ^4^ ^]^

It is important to note that in stage 2 (Figure 4) {220}β deforms plastically prior to the activation of twinning in stage 3, and yields before the onset of the latter effect. This points to a decisive role of the β interfaces in triggering the activation of twinning, since twins usually start as embryos at grain boundaries, where stress concentrations and source defects (e.g., dislocation pile‐up) are predominately located.^[^ ^45^ ^]^ However, differently to that of coarser lamellar α + β microstructures, the deformation behavior in the current investigations is mainly imposed by α. Thus, the introduction of β interfaces can be regarded as a source of nucleation sites for twinning. This is also in line with previous investigations of coarser microstructures suggesting the nucleation of twins within α/β interfaces as a consequence of local deformation and dislocation activity.^[^ ^46^ ^]^ In contrast, mechanical twinning is not a dominant deformation mechanism in the majority of the as‐built conditions of Ti–6Al–4V obtained by LBPF. These are usually martensitic microstructures presenting a lack of β interfaces. The approach taken in this work reveals that optimization of the IHT allows for exploiting the interface behavior which can activate mechanical twinning and consequently improve both the strength and ductility of this alloy.

Slip is the typical deformation mechanism in the Ti–6Al–4V alloy, while twinning‐induced plasticity has been rarely observed. In such cases, it was reported for post‐mortem analysis of much coarser α + β microstructures as well as conditions undergoing severe plastic deformation and high strain rates.^[^ ^17^, ^46^, ^47^ ^]^ In this work, in situ HEXRD allowed for elucidating in real time the mechanism of twinning formation in the fine‐grained microstructure of Ti–6Al–4V obtained by LPBF. Owing to the anisotropy of this alloy, the tuning of the initial texture is important in order to take advantage of the combined effects of slip and twinning. Our recent investigations evaluating the role of texture, show that this can lead to a different activation sequence of deformation mechanisms.^[^ ^48^ ^]^ In the present study, the introduction of ductile β interfaces in a microstructure with favorable texture leads to the activation of twinning as an alternative deformation mechanism which can be exploited to improve the strength–ductility trade‐off of Ti–6Al–4V.

## 4 Conclusion

In summary, this work discovers that the grain size effect, which usually hinders the activation of deformation twinning in fine‐grain hcp microstructures, can be overcome by the introduction of ductile nano‐layer interfaces. In situ synchrotron HEXRD enables a continuous tracking of the deformation kinetics including the activation of twinning‐induced plasticity and its evolution. The findings obtained show that the typical main deformation by slip in the hcp matrix of Ti–6Al–4V can be complemented by TWIP by the introduction of the more ductile phase at the grain interfaces, that is, bcc β nanolayers. Thus, the strength–ductility trade‐off of the alloy is remarkably improved. Modifying the grain interfaces for promoting twinning in small grain hcp microstructures opens up new strategies to develop new damage tolerant alloys.

## 5 Experimental Section

### Laser Powder Bed Fusion

A Ti–6Al–4V (wt%) grade 23 powder alloy was processed by laser powder bed fusion (LBPF) in argon 5.0 atmosphere using a SLM280HL machine (SLM solutions GmbH). The chemical composition of the powder according to the specification provided by AP&C is 0.11 wt% O, 6.39 wt% Al, 3.80 wt% V, and 0.19 wt% Fe. Oxygen was determined by the powder provider AP&C using inert gas fusion following ASTM E1409 and the metallic elements were determined according to ASTM E2371 by direct current plasma emission spectroscopy. The atmosphere of the LPBF chamber was carefully controlled to avoid O pick‐up during processing. The temperature of the building platform was set to 200 °C. The powder alloy was produced by gas atomization and consisted of spherical particles of size distribution D10 = 22 µm, D50 = 34 µm, and D90 = 46 µm measured by the standard method ASTM B822. Cylindric samples were built using a chess scanning strategy. The laser pattern for the bulk material consisted of chess domains, where the scanning vectors between chess domains present a relative rotation of 90°.^[^ ^41^ ^]^ The following are the SLM processing parameters employed: laser power = 250 W, scanning velocity = 1000 mm s^−1^, hatch distance = 40 µm, focal offset distance = 0 mm, and layer thickness = 30 µm, resulting in a volume energy density = 208.3 J mm^−3^. This strategy aims at promoting partial decomposition of α′ martensite in order to obtain fine α grains decorated by β nanolayers during LPBF.^[^ ^42^ ^]^ For compression testing, cylindrical samples built with their longitudinal axis parallel to the build plate of the LPBF machine were considered owing to their main texture component with the hcp *c*‐axis oriented parallel to the compression direction. These samples were machined down to a 3.0 mm diameter and 5.5 mm length. This permitted to remove the supports and the poor surface roughness quality of the as‐built samples.

### In Situ High Energy Synchrotron X‐Ray Diffraction

In situ high energy synchrotron X‐ray diffraction (HEXRD) was performed during uniaxial compression in transmission mode (thickness = 3 mm) at the beamline P07‐HEMS in PETRA III (Deutsches Elektronen‐Synchrotron, DESY, Hamburg, Germany).^[^ ^49^ ^]^ The energy of the X‐ray beam and the wavelength used were 100 keV and 0.124 Å, respectively. The slit‐aperture size of the beam was 1 × 1 mm^2^. The incident X‐ray beam was positioned at the center of the samples before and during deformation. A PerkinElmer XRD 1621 acquired the Debye–Scherrer rings from the samples during deformation within intervals of 0.5 s per image.

The compression test was carried out at room temperature using a modified dilatometer Bähr 805A/D equipped with a deformation unit.^[^ ^50^ ^]^ The cylindrical samples were compressed at a strain rate of 0.002 s^−1^. No buckling of the samples was observed during deformation. An illustrative diagram of the experimental setup is shown in ref. [51].

The acquired Debye–Scherrer rings were converted into Cartesian coordinates (azimuth angle ψ, 2θ) in order to investigate the evolution of the diffraction images obtained in situ during deformation. Then, the intensity sum of Bragg reflections was projected on the azimuthal axis using the software ImageJ.^[^ ^52^ ^]^

The software Maud^[^ ^53^ ^]^ was employed for quantitative analysis of the diffraction patterns by the Rietveld method. The instrumental parameters of the HEXRD setup were obtained from LaB6 powder standard. The lattice strains of the compression samples were calculated according to Equation (1):

(1) $$\varepsilon_{\text{hkl}}=\frac{d_{\text{hkl}}^{i}-d_{\text{hkl}}^{0}}{d_{\text{hkl}}^{0}}$$

where ε_hkl_ is the lattice strain of a {hkl} plane family, *d* ^0^_hkl_ and *d* ^i^_hkl_ are the interplanar distances for a {hkl} family before deformation and at a deformation step *i*, respectively. To this purpose, cake portions of 10° in the load direction were taken from the Debye–Scherrer rings. Further details of this methodology are provided in ref. [45]. The 2θ variations of individual {hkl} reflections were considered for this analysis. An extended Williams–Imhof–Matthies–Vinel algorithm (E‐WIMV)^[^ ^53^ ^]^ combined with a moment pole stress model integrated in MAUD were used for Rietveld and texture analysis.

### Microstructure Characterization

Scanning electron microscopy (SEM) in backscattered electron mode (BSE) and transmission electron microscopy (TEM) were used to characterize the microstructure of the samples in the conditions as‐built and after uniaxial compression up to failure. For SEM, the specimens were prepared by grinding and polishing using a TegraPol machine. SEM was performed by employing a FEI Helios‐Nanolab600i dual‐beam (electron and Ga^+^) setup which was also used for the preparation of a TEM lamella by focused ion beam (FIB). The latter was extracted from the alloy's polished surface by FIB milling. Before that, a platinum layer of 2 µm was deposited on the region of interest. The sample was prepared using the in situ lift‐out technique according to the procedure described in ref. [54]. A final ion polishing step was performed up to electron transparency at a low voltage of 5 kV and at 14 pA in order to avoid Ga^+^ implantation and minimize FIB induced damage. This operation was performed with the Omniprobe lift‐out‐grid used for TEM. The sample investigated, of section ≈12 × 12 µm^2^ and thickness ≈100nm, was prepared uniformly thin, that is, permitting a homogeneous penetration of the electron beam. The samples were examined with a Philips Tecnai F30 microscope operated at 300 keV. Energy dispersive spectroscopy (EDX) was performed using an EDAX Apollo XLTW EDX detector implemented in this device. Theoretical Schmid factors were calculated using the MTEX software^[^ ^55^ ^]^ and the crystal lattice parameters obtained from Rietveld refinements.

## Conflict of Interest

The authors declare no conflict of interest.

## Supporting information

Supporting Information

## Data Availability

Research data are not shared.
