# Supplementary material for: Interface‐Mediated Twinning‐Induced Plasticity in a Fine Hexagonal Microstructure Generated by Additive Manufacturing
Source: Adv Mater. 2021 Oct 19;33(52):2105096. doi: 10.1002/adma.202105096 (PMC11468652; doi:10.1002/adma.202105096)
Supplement: Supplementary file 1 — Supporting Information [file ADMA-33-2105096-s001.pdf]

ADVANCED MATERIALS

Supporting Information

for *Adv. Mater.,* DOI: 10.1002/adma.202105096

Interface-Mediated Twinning-Induced Plasticity in a Fine Hexagonal Microstructure Generated by Additive Manufacturing

Pere Barriobero-Vila, Juan Manuel Vallejos, Joachim Gussone, Jan Haubrich, Klemens Kelm, Andreas Stark, Norbert Schell, and Guillermo Requena*

Supporting Information

Interface-mediated twinning-induced plasticity in a fine hexagonal microstructure

Pere Barriobero-Vila, Juan Manuel Vallejos, Joachim Gussone, Jan Haubrich, Klemens Kelm, Andreas Stark, Norbert Schell, Guillermo Requena

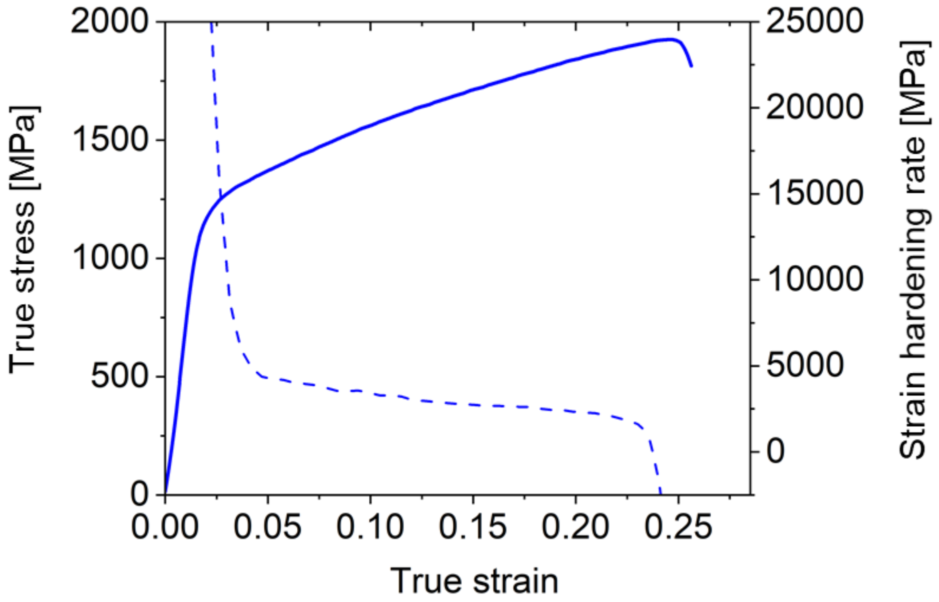

Figure S1. Curves for the evolution of the true stress-strain and strain hardening rate (θ=dσ/dε) obtained during uniaxial compression of the studied laser-powder bed fusion Ti-6Al-4V alloy represented by continuous and dashed lines, respectively.

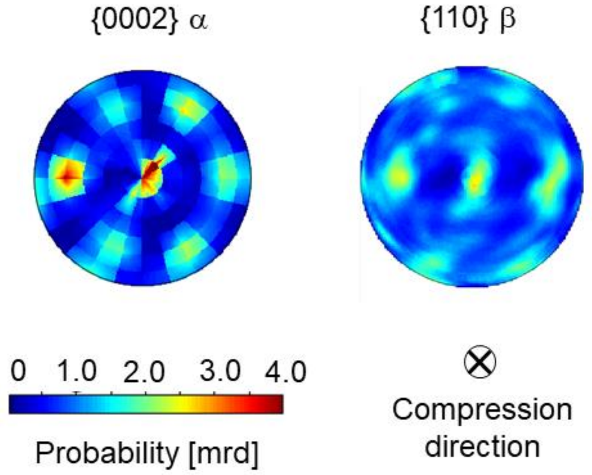

Figure S2. Pole figures of {0002}α and {110}β obtained from the bulk material by in situ HEXRD (gauge volume 1×1×3.5 mm^3) for the LPBF as-built condition.
